# Unusual case of paraplegia

**DOI:** 10.4103/0972-2327.56321

**Published:** 2009

**Authors:** Prem Amalraj, S. Syamlal

**Affiliations:** Department of Neurology, Kerala Institute Medical Science, Thiruvananthapuram, Kerala, India

**Keywords:** Acute myeloid leukemia, chloroma, granulocytic sarcoma

## Abstract

Paraplegia due to a spinal cord epidural mass is an extremely rare presentation of undiagnosed leukemia. We are reporting a case of 14-year-old girl, who presented with paraplegia due to thoracic epidural mass, as the initial presenting manifestation of acute myeloid leukemia. Granulocytic sarcoma or chloroma should be considered in the differential diagnosis of an epidural mass in patients with or with out leukemia granulocytic sarcoma, which are rare extramedullary tumor-like proliferation of myelogenous precursor cells that may de novo precede acute leukemia or coincide with the first manifestation or relapse of acute myeloid leukemia.

## Introduction

Paraplegia due to chloroma is an extremely rare presenting neurological manifestation in myeloid leukemia.[1] Chloromas are most frequently seen in acute myeloid leukemia (AML).[2] Also, it may signal the onset of accelerated phase of chronic myeloid leukemia or blastic transformation of a myeloproliferative disorder.[3] Granulocytic sarcoma should be considered in the differential diagnosis of an epidural mass in patients with or without leukemia as granulocytic sarcoma represent a diagnostic challenge, particularly those occurring in patients with out evidence of systemic disease

## Case Report

A 14-year-old schoolgirl presented with progressive weakness of both lower limbs of two weeks duration associated with numbness of both lower limbs, which ascended up to lower chest region. She was having back pain for the last one month for which she consulted local hospital. Later, she developed bladder symptoms as hesitancy, and urgency for the last one week. No upper limb weakness or cranial nerve symptoms. No h/o fever, significant weight loss, or contact with tuberculosis was diagnosed.

On examination, she was thin built and vitals were normal. Cranial nerve examinations including fundus were normal. Upper limb powers were normal while in lower limbs she had grade 1 power in both lower limbs. Tendon reflexes were absent in lower limbs with bilateral extensor plantar response. She had a sensory level at D4 with spinal tenderness at mid thoracic region. Other system examinations were normal.

**Figure 1:**
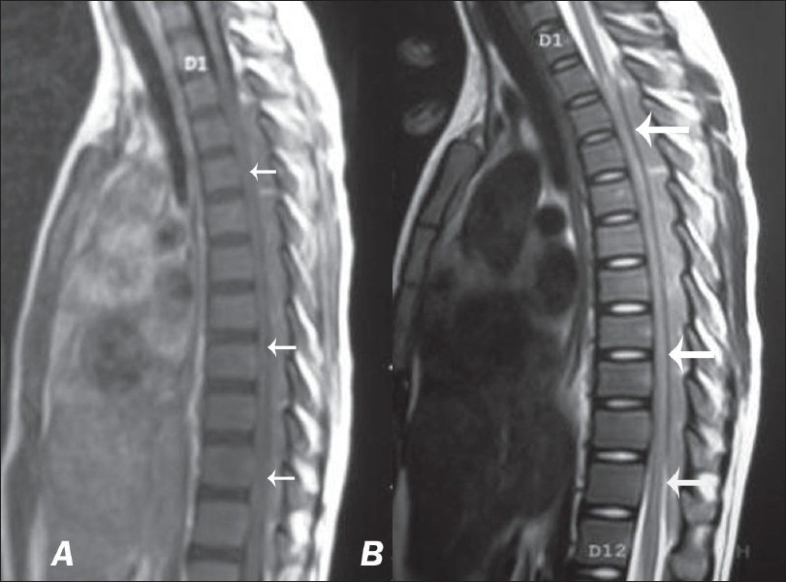
MRI of the dorsal spine showing an epidural mass that is hypointense in T1WI (figure 1A) and hyperintense in T2WI with a distinct line of dural demarcation (see arrows) in figure 1B

**Figure 2:**
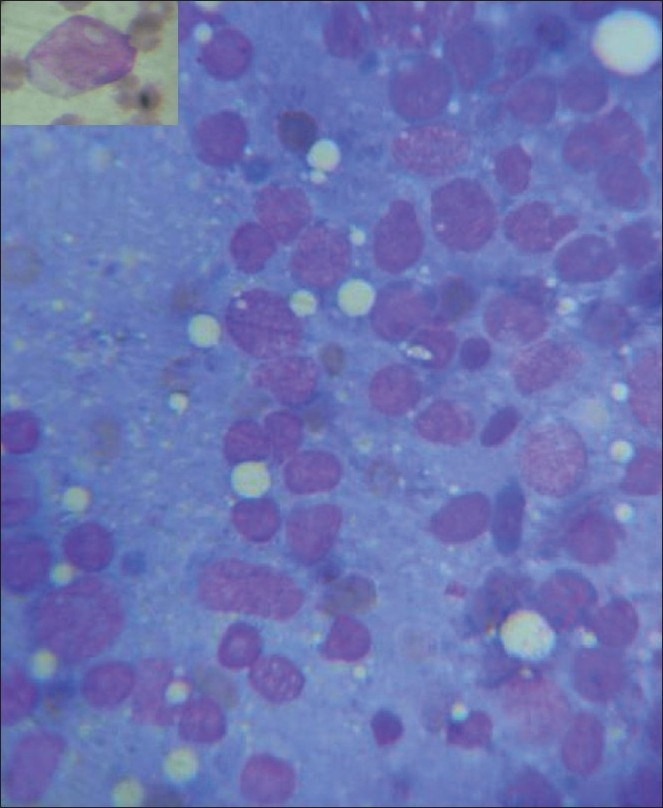
Peripheral smear showing blast cells (inset) and bone marrow showing blast cells s/o acute myeloid leukemea

Investigations done showed hemoglobin 13 gm%, total count 8,400, polymorph 60%, and lymph 40%, with high erythrocyte sedimentation rate (ESR) of 130. Chest x-ray was normal. Lumbar Cerebrospinal fluid (CSF) study showed: Total count of 104 cells/mm with 90% lymph, protein 802 mg/dl, and glucose 56 mg/dl. Gram stain, AFB stain, cultures, and cytology for malignant cells were negative. Magnetic resonance imaging (MRI) showed extensive epidural lesion extending from D3 to D11, hypointense in T1 [Figure 1A], hyperintense in T2 [Figure 1B], dorsally compressing the spinal cord. With this clinical picture and investigations considering the possibility of tuberculosis, patient was started on antitubercular treatment with steroids. CSF TB PCR report came as negative and there was no significant improvement in clinical picture. Then we reassessed the case and peripheral smear, serum ACE levels were send and planned CT guided biopsy. Then peripheral smear [Figure 2 inset] report came as RBC mild hypochromic microcytic, WBC count normal predominantly blast cells with high nuclear cytoplasmic ratio, moderate cytoplasm, hyperchromatic nuclei with multiple nucleoli, platelet count decreased. Bone marrow [Figure 2] test revealed marrow elements completely replaced with blast cells having high nuclear cytoplasmic ratio, moderate amount of eosinophilic cytoplasm, and hyperchromatic nuclei with multiple nucleoli s/o promyelocytic leukemia AML M3. Considering the possibility of granulocytic sarcoma as the cause of paraplegia in acute myeloid leukemia, she was started on local radiotherapy and chemotherapy. Her paraplegia improved with radiotherapy and chemotherapy.

## Discussion

Paraplegia, as the initial presenting feature of acute myeloid leukemia, is very rare. Chloroma should be considered in the differential diagnosis of any epidural mass in patients with or without leukemia. Granulocytic sarcoma occurs in 3 to 9% of AML cases.[1] Rarely chloroma can occur alone without peripheral blood or bone marrow evidence of leukemia. The interval between initial diagnosis and the onset of acute leukemia has been reported between 1 and 49 months (average, 10 months).[3] The most common sites of chloroma are: bone, skin, and lymph nodes; and in bones orbit: sacrum, spine, and ribs.[3] Macroscopically, chloromas are green in appearance due to the myeloperoxidase in the leukemia cells and fades when exposed to air.[4] The origin of the tumor is in the bone marrow blast cells and migrates to extraosseus locations through haversian canals.[5]

In our patient, paraplegia due to chloroma as the initial presenting feature of AML, is very rare. MRI usually shows epidural mass lesion compressing the cord, isointense with cord in T1, and hyperintense in T2-weighted images. The differential diagnosis includes epidural abscess, metastasis, and lymphoma deposits. Lymphoma usually shows epidural extension of tumor directly from vertebral body and inhomogeneous hyperintense signals in T2 images of vertebral body due to marrow infiltration, which is not seen in chloroma. CSF cytology for malignant cells will be negative in extradural compressive myelopathy due to chloroma as in our case.

Granulocytic sarcomas are usually radiosensitive and are often treated with local radiotherapy and chemotherapy.[6,7] Newly diagnosed patients with isolated granulocytic sarcomas usually treated with aggressive chemotherapy as if they have acute myelogenous leukemia; cures are not attained with radiation therapy alone.[6] Surgery is generally preferred for cases of acute spinal cord compression in cases with out systemic evidence of leukemia.[7,8] Early diagnosis followed by appropriate combined chemotherapy and radiation may obviate surgical intervention and eventually prevent leukemic transformation. Chloroma, though rare, should be considered in the differential diagnosis of any epidural mass lesion and peripheral smear examination and bone marrow study, if necessary, should be done in all cases.
